# A Study on the Effects of Deep Eutectic Solvent (Chcl-Eg) Pretreatment on the Mechanical and Dimensional Stability Properties of Densified Chinese Fir

**DOI:** 10.3390/ma19163527

**Published:** 2026-08-20

**Authors:** Yun Qian, Shiyu Liu, Yalan Qian, Yunyan Peng, Wenbo Che, Haili Chen, Youming Yu, Wei Zheng

**Affiliations:** 1 College of Chemistry and Materials Engineering, Zhejiang A&F University, Hangzhou 311300, China; qy2023104041014@stu.zafu.edu.cn (Y.Q.); liu13387849277@163.com (S.L.); 17561845990@163.com (Y.Q.); pengyunyan@zafu.edu.cn (Y.P.); chewenbo@zafu.edu.cn (W.C.); hchen14@zafu.edu.cn (H.C.); 2 Jiyang College, Zhejiang Agriculture and Forestry University, Zhuji 311800, China

**Keywords:** Chinese fir, deep eutectic solvent, wood densification, dimensional stability, mechanical properties

## Abstract

Hot-pressing densification is an effective method to improve the physical and mechanical properties of fast-growing wood, but it typically faces challenges such as moisture-induced rebound and brittle fracture of cell walls. This work proposed a strategy for preparing densified Chinese fir (DCF) via deep eutectic solvent (DES) pretreatment. The effects of pretreatment conditions (i.e., DES concentration, treatment temperature, and treatment time) and physical compression ratio on the dimensional stability, mechanical properties, and microstructure of DCF were systematically investigated. The results demonstrated that under the best-performing conditions within the investigated range conditions (10 wt% DES concentration, treatment at 100 °C for 8 h, and 50% compression ratio), the modified fir (viz., DCF) exhibited substantial improvements compared to NW in terms of modulus of rupture (147.8 MPa, a 163.5% increase), modulus of elasticity (8622 MPa, a 71.1% increase), compressive strength along the grain (81.8 MPa, a 138.5% increase), and Shore D hardness (70.6, an increase of 83.9%). Furthermore, the moisture-induced rebound rate was reduced to 0.77%, indicating that the dimensional stability of DCF was effectively enhanced. Microstructural and chemical characterization revealed that DES partially degraded part of the amorphous hemicelluloses, thereby plasticizing the cell wall and enabling cells to undergo flexible folding and dense closure during the hot-pressing process while largely retaining the main cellulose crystalline skeleton. The present work provides a feasible route for converting fast-growing plantation wood into structural materials.

## 1. Introduction

Chinese fir (CF), *Cunninghamia lanceolata* (Lamb.) Hook., is an important fast-growing tree species for artificial forests in China. Due to its rapid growth and straight grain, it is widely used to alleviate the growing demand for natural forest wood [1,2]. Nevertheless, short rotation periods typically result in CF having characteristics such as high porosity, large cell lumina, and thin cell walls, leading to a low basic density (0.30–0.42 g/cm^3^) and weak mechanical strength. Notably, these inherent defects severely limit its application in structural engineering and high-end manufacturing fields. To overcome these challenges, thermal densification is widely recognized as an effective physical modification strategy. During this process, applying heat and mechanical compression substantially reduces the pore volume in wood, causing cell walls to collapse and pack tightly together, thereby increasing the density and mechanical load-bearing capacity [3,4].

Although thermal densification can enhance the physical strength of wood, it remains subject to the following key challenges: (i) without sufficient softening, the rigid cell walls of untreated wood are prone to localized brittle fracture or microcracks under extreme compressive stress [5,6]; (ii) Second, during physical compression, the cellulose macromolecular chains undergo forced deformation and store a large amount of elastic potential energy. When densified wood is exposed to a humid environment, moisture easily penetrates the cell walls and destroys the newly formed hydrogen bonds. This triggers the release of internal stresses, inevitably leading to severe dimensional rebound and compromising structural stability [7]. Based on this, chemical pretreatment methods such as alkali solutions and traditional ionic liquids have been introduced to partially remove the matrix polymer before pressing to soften the wood. Notwithstanding this, these traditional methods are often limited by environmental pollution, high costs, or the non-selective over-degradation of the cellulose skeleton, negatively affecting the inherent mechanical strength of the wood [8,9].

Considerable research has been conducted on wood densification modification over recent years. Conventional thermal densification and thermo-hydro-mechanical treatment have been widely shown to effectively improve wood density and mechanical strength by reducing internal porosity, but they still fail to fundamentally address the two core problems of moisture-induced dimensional rebound and cell-wall brittle fracture. To improve the shape-fixation effect of densified wood, researchers have proposed various chemical pretreatment strategies. Alkaline treatment and ionic liquid pretreatment can enhance the plastic deformation capacity of wood cell walls to a certain extent, but they generally have shortcomings such as environmental pollution, high cost, or non-selective damage to the cellulose crystal skeleton, which will impair the inherent mechanical framework of wood. In recent years, deep eutectic solvents have shown great application potential in wood modification due to their relatively greener and milder characteristics, but most relevant studies focus on hardwood species with relatively loose anatomical structures. There is still a lack of systematic research on the process optimization, performance regulation mechanism, and machining adaptability of DES-assisted densification for typical fast-growing softwood represented by Chinese fir.

Recently, deep eutectic solvents (DES) have attracted widespread attention as emerging tunable solvents with green and sustainable potential for modifying lignocellulosic biomass [10]. As is known, DES typically consists of a hydrogen-bond acceptor and a hydrogen-bond donor, offering considerable advantages such as simple preparation, low toxicity, and biodegradability. Specifically, choline chloride-based DES systems (e.g., choline chloride-ethylene glycol) can effectively break the bonds within the lignin–carbohydrate complex, partially removing part of the amorphous hemicelluloses and lignin while largely retaining the robust crystalline cellulose framework; thus, they exhibit favorable component-modulation capabilities [11,12,13,14]. Notably, this targeted deconstruction not only effectively plasticizes the cell wall, reducing deformation resistance, but also minimizes the primary source of internal stress. For instance, Ran et al. and Gondaliya et al. demonstrated that DES pretreatment in hardwood species facilitates flexible cell wall folding and hydrogen-bond reconstruction during compression, effectively mitigating moisture-induced dimensional rebound [15,16].

Based on this, the present work proposed a strategy to prepare dimensionally stable densified Chinese fir (DCF) through sequential choline chloride–ethylene glycol DES pretreatment followed by hot pressing [17,18]. To comprehensively understand the mechanism of action for DES, a systematic evaluation was conducted to determine the effects of critical process parameters (i.e., DES concentration, treatment temperature, treatment time, and physical compression ratio) on the mass loss and dimensional stability (moisture-induced rebound and thickness expansion) of DCF. Additionally, the laws governing microstructural reconstruction, chemical structure evolution, and improvements in mechanical properties (modulus of rupture, elastic modulus, compressive strength, and surface hardness) were investigated across multiple scales. This work aims to elucidate the synergistic mechanisms underlying DES-induced plasticization and shape locking, thereby providing a practical and relatively eco-friendly technical route for the utilization of fast-growing plantation wood.

## 2. Experimental Section

### 2.1. Materials

Chinese fir boards (purchased from Alibaba, Hangzhou, China) free of defects such as cracks, deformation, insect damage, mildew, or decay were selected as raw materials. The selected wood samples consisted primarily of heartwood and were processed into uniform wood panels measuring 190 mm (longitudinal) × 80 mm (tangential) × 10/12 mm (radial). The samples were conditioned at 20 ± 2 °C and 65 ± 5% relative humidity to ensure a moisture content of approximately 12%, and these untreated logs were used as the control group (Natural Wood, NW). The experimental reagents, 98% reagent-grade choline chloride and 99% ethylene glycol, were both purchased from Aladdin Biochemical Technology Co., Ltd., Shanghai, China.

### 2.2. DES Pretreatment and Hot-Pressing Densification

Choline chloride and ethylene glycol were mixed in a 1:2 molar ratio and heated with stirring until a uniform, transparent DES stock solution was formed; the solution was then diluted with deionized water to the specified mass fraction. The Chinese fir samples were completely immersed in the DES solution and pretreated at the designated temperature and time. After treatment, the samples were repeatedly washed with deionized water until they reached neutrality, then slowly dried at 40–50 °C and conditioned to a moisture content of 25–30% before undergoing hot-pressing densification [19]. Specifically, the hot-pressing densification was conducted using a laboratory hot press at 140 °C. To precisely achieve the target compression ratios of 20%, 30%, 40%, and 50%, corresponding thickness gauges (mechanical stops) were employed between the press plates to control the final thickness of the samples. The press was gradually closed, and a pressure of approximately 5 MPa (sufficient to compress the wood to the target thickness) was applied and maintained for 30 min. Subsequently, to permanently fix the compressive deformation and prevent immediate spring-back, the densified samples were cooled under pressure to room temperature before the press was opened. Finally, all the densified samples were stored in a drying cabinet for post-press conditioning to relieve internal residual stresses before characterization. Moreover, Single-factor and control-variable methods were used in this work to investigate the effects of the DES concentration (0, 5, 10, 15, and 20 wt%), treatment temperature (60, 80, 100, and 120 °C), treatment time (2, 4, 6, and 8 h), and physical compression ratio (20, 30, 40, and 50%) on the dimensional stability, mechanical properties, and microstructure of DCF. It should be explicitly noted that the group treated with a 0% DES concentration serves as the pure water-treated densified control group. These samples underwent the same hot-pressing densification process at the corresponding compression ratios. Comparing this 0% DES control group with the DES-treated groups allows for the clear separation of the chemical modification effects induced by the DES from the mechanical effects of physical densification.

### 2.3. Mass Loss Rate

The weight percentage loss after DES pretreatment was calculated based on the change in absolute dry mass, as shown in Equation (1):

(1) $$W_{L}\;=\;\frac{m_{0}-m_{t}}{m_{0}}\;\times \;100\%$$

where *W_L_*, *m*_0_, and *m_t_* are the weight percentage loss of samples (%), the initial absolute dry mass of the sample before treatment (g), and the absolute dry mass after DES pretreatment (g), respectively. For each pretreatment condition, eight independent specimens were tested to calculate the average mass loss.

### 2.4. Density Profile Distribution Test

To evaluate the degree of compaction and the density gradient along the thickness direction of Chinese fir during the densification process, an X-ray density profile analyzer (DENSE-LAB, EWS, Hameln, Germany) was used. A sample with a cross-section of 50 mm × 50 mm was prepared and dried at 60 °C until constant weight to minimize the effect of moisture on X-ray attenuation. Of these, the X-ray beam scans layer by layer along the thickness (compression) direction for the samples using a 0.01 mm scan step.

### 2.5. Dimensional Stability

Dimensional stability was determined on absolutely dry and densified samples. The test method refers to ISO 13061-15:2025 (Physical and mechanical properties of wood—Test methods for small clear wood specimens—Part 15: Determination of radial and tangential swelling) [20]. Specifically, the initial thickness of the samples in the radial (compression) direction was first measured. Subsequently, the samples were conditioned in a constant-temperature and constant-humidity chamber at 20 ± 2 °C and 65 ± 5% relative humidity until a constant weight was reached (defined as a mass change of less than 0.2% over a 2 h interval), at which stage their thickness was measured to calculate the moisture-absorption rebound rate (*SR*). To measure the thickness swelling ratio (*TS*) after water absorption, another batch of absolutely dry samples was fully immersed in deionized water for 24 h to determine the change in thickness. Eight independent specimens were evaluated for each condition to determine the average *SR* and *TS* values. The *SR* and *TS* parameters were calculated using Equations (2) and (3).

(2) $$\mathrm{SR}=\frac{t_{2}-t_{1}}{t_{0}-t_{1}}\;\times \;100\%$$

(3) $$\mathrm{TS}=\frac{h_{2}-h_{1}}{h_{1}}\;\times \;100\%$$

where t_0_, t_1_, and t_2_ represent the absolute dry thickness of NW (mm), the absolute dry thickness of DCF (mm), and the radial thickness of DCF after 24 h of moisture absorption equilibrium (mm), respectively. h_1_ is the absolute dry thickness of the samples before water immersion (mm); h_2_ denotes the wet thickness of the samples after 24 h of water immersion (mm).

### 2.6. Mechanical Properties and Hardness

The modulus of rupture (MOR) and modulus of elasticity (MOE) of the samples were evaluated according to the international standard ISO 13061-4:2014, using a universal mechanical testing machine (Instron 5967, Instron, Norwood, MA, USA) for a three-point bending test, with a loading rate set at 2.0 mm/min [21]. Moreover, the test for compressive strength (CS) along the grain was performed following the international standard ISO 13061-17:2017, employing a loading rate of 1.0 mm/min [22]. The surface hardness of the samples was determined using a Shore D hardness tester (LX-D, HANDPI, Yueqing, China). Specifically, the indenter was applied perpendicularly to the tangential cross-section of the samples, with eight test points randomly selected for each sample and the average value calculated. For both bending and compressive tests, eight independent specimens were prepared and evaluated for each condition.

### 2.7. Rotary-Cut Surface Micro-Topography

The DCF was machined into a cylinder simulating the radial dimensions of a standard pencil, and spiral micro-peeling sampling was performed using a micro-cone former. Subsequently, the detached fan-shaped flakes were collected, precisely trimmed into regular 4 mm × 4 mm samples, and mounted flat on a microscope slide. Finally, a 3D deep-field microscope (VHX-1000, Keyence Corporation, Osaka, Japan) was used to perform high-precision quantitative characterization of the surface topography and roughness of these micro-samples. It should be noted that due to the intrinsic spiral curvature and minute size of the micro-peeled fan-shaped flakes, standard flat-plane macroscopic roughness measurements could not be performed. Instead, the maximum height difference within the selected micro-areas was extracted as a localized, semi-quantitative indicator to evaluate cutting uniformity.

### 2.8. Material Characterization

The TM3030 scanning electron microscope (SEM) (Hitachi High-Technologies Corporation, Tokyo, Japan) was used to observe the micro-morphology of the materials. The chemical properties of the samples were detected by the Fourier transform infrared spectrometer (Nicolet iS50, Thermo Fisher Scientific, Waltham, MA, USA). Additionally, the X-ray diffraction (XRD) of samples was measured by an X-ray diffractometer (D8 ADVANCE, Bruker Corporation, Karlsruhe, Germany). The scanning speed and range were 5 °/min and 5–45° for XRD measurement. Notably, the relative crystallinity index (*CrI*) was calculated according to the Segal formula (4):

(4) $$C_{r}I=\frac{\left(I_{002}-I_{\mathrm{am}}\right)}{I_{002}}\times 100\%$$

where I_002_ represents the maximum intensity of the (002) lattice diffraction angle; I_am_ refers to the scattering intensity of amorphous diffraction at ~18° in the 2θ angle.

### 2.9. Statistical Analysis

All quantitative data in this study were obtained from eight independent replicate experiments. The results are expressed as the mean value ± standard deviation (SD) to demonstrate data dispersion. The error bars in all graphical representations represent the SD. The macroscopic trends and physical differences between the untreated and treated groups were evaluated based on these descriptive statistics using Origin 2024 software (OriginLab Corporation, Northampton, MA, USA).

It should be noted that the current study primarily relies on descriptive statistics to evaluate the macroscopic trends of the treatment parameters. While this approach provides an intuitive representation of the overall variations under different conditions, the lack of inferential statistical analysis (e.g., ANOVA) is a limitation of this exploratory study, which will be systematically addressed in future scale-up research.

## 3. Results and Discussion

### 3.1. The Effect of DES Pretreatment on the Mass-Loss Rate

**Figure 1:**
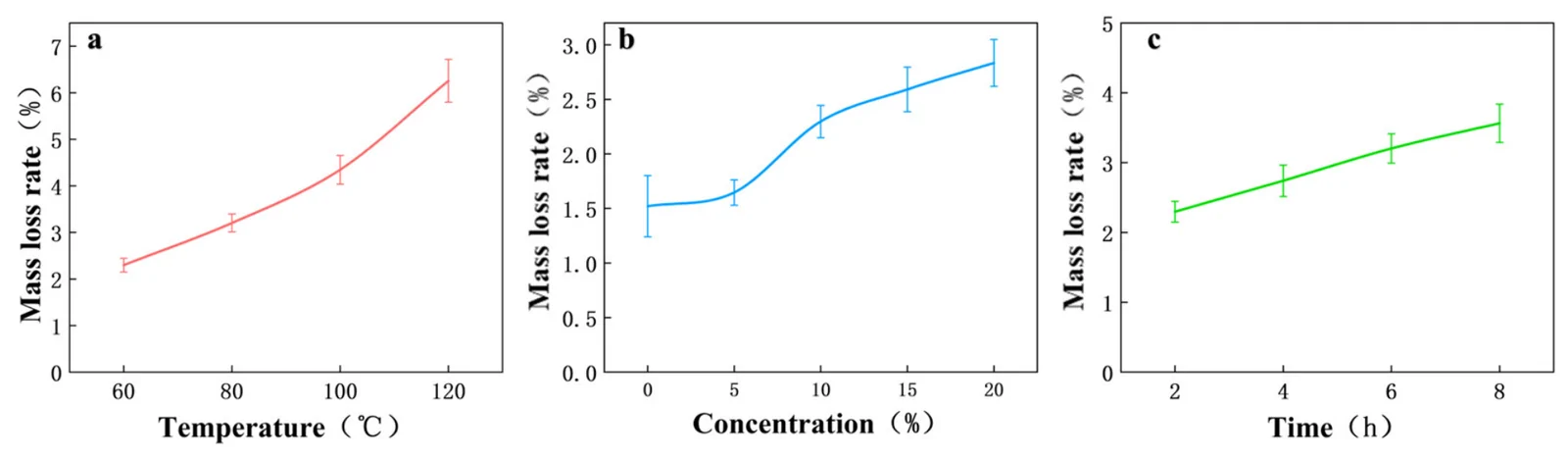
Mass-loss rates of the samples at different (**a**) pretreatment temperatures, (**b**) DES concentrations, and (**c**) pretreatment times. (Note: The lines connecting the data points are intended to guide the eye. Error bars represent standard deviations).

As is known, the mass-loss rate directly reflects the extent to which DES pretreatment affects the components of the wood cell wall. With increasing DES concentration, treatment temperature, and treatment time, the mass-loss rate of Chinese fir generally showed an upward trend yet remained at a low level (≤6.26%) under all test conditions. When the DES concentration reached 10 wt% and the treatment time was 2 h, the mass-loss rate markedly increased from 2.30% at 60 °C to 6.26% at 120 °C (Figure 1a), indicating that temperature is a critical thermodynamic factor driving the leaching and structural rearrangement of the amorphous components within lignocellulose. In contrast, under the reference condition of 60 °C and 2 h, when the DES concentration was increased from 0 to 20 wt%, the mass-loss rate rose only slightly from 1.52% to 2.83% (Figure 1b). Similarly, the mass-loss rate increased from 2.30% to 3.56% as the treatment time was extended from 2 h to 8 h (Figure 1c). This gentle growth trend exhibits typical and mild diffusion-controlled characteristics, indicating that the choline chloride–ethylene glycol aqueous solution system utilized in this work is not a strong delignification system but rather tends toward a mild cell wall plasticization system [23].

Notably, the control of mass loss within a low range is particularly crucial for preparing high-performance densified wood. For instance, excessive removal of lignin and hemicelluloses severely weakens the mechanical framework of the cell wall, leading to a high susceptibility to microcracks and interlaminar delamination during high-temperature hot pressing; conversely, insufficient plasticization prevents effective reduction of the stress yield recorded in the cell wall, facilitating brittle fracture of the cells during compression. In this work, the DES system caused only limited mass loss in the samples, thereby achieving precise control over the amorphous components and effective softening of the cell wall, which provided an ideal microstructural foundation for the subsequent flexible folding of guard cells, the tight closure of cell cavities, and the reconstruction of interfacial hydrogen bonds [15,24]. In other words, the core advantage of this method lies not in intense delignification, but in achieving an excellent balance between low-damage plasticization and mechanical framework retention.

### 3.2. The Enhancement Effect of DES Pretreatment on Mechanical Properties

Sequential DES pretreatment-hot pressing significantly improved the mechanical properties recorded in the samples, as listed in Table 1.

**Figure 2:**
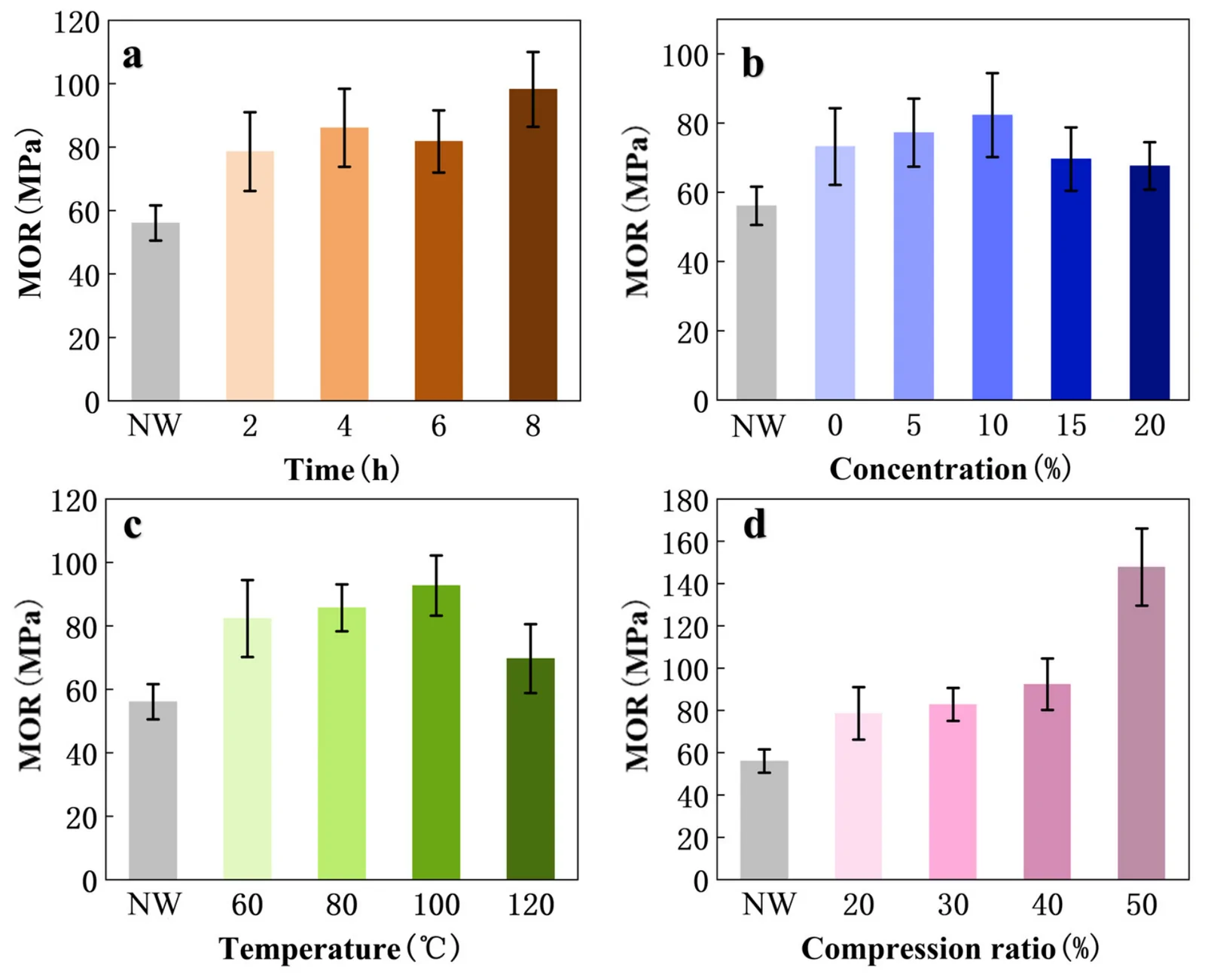
Modulus of rupture of NW and DCF at different (**a**) treatment times, (**b**) DES concentrations, (**c**) treatment temperatures, and (**d**) compression ratios. (Note: Error bars represent standard deviations).

**Figure 3:**
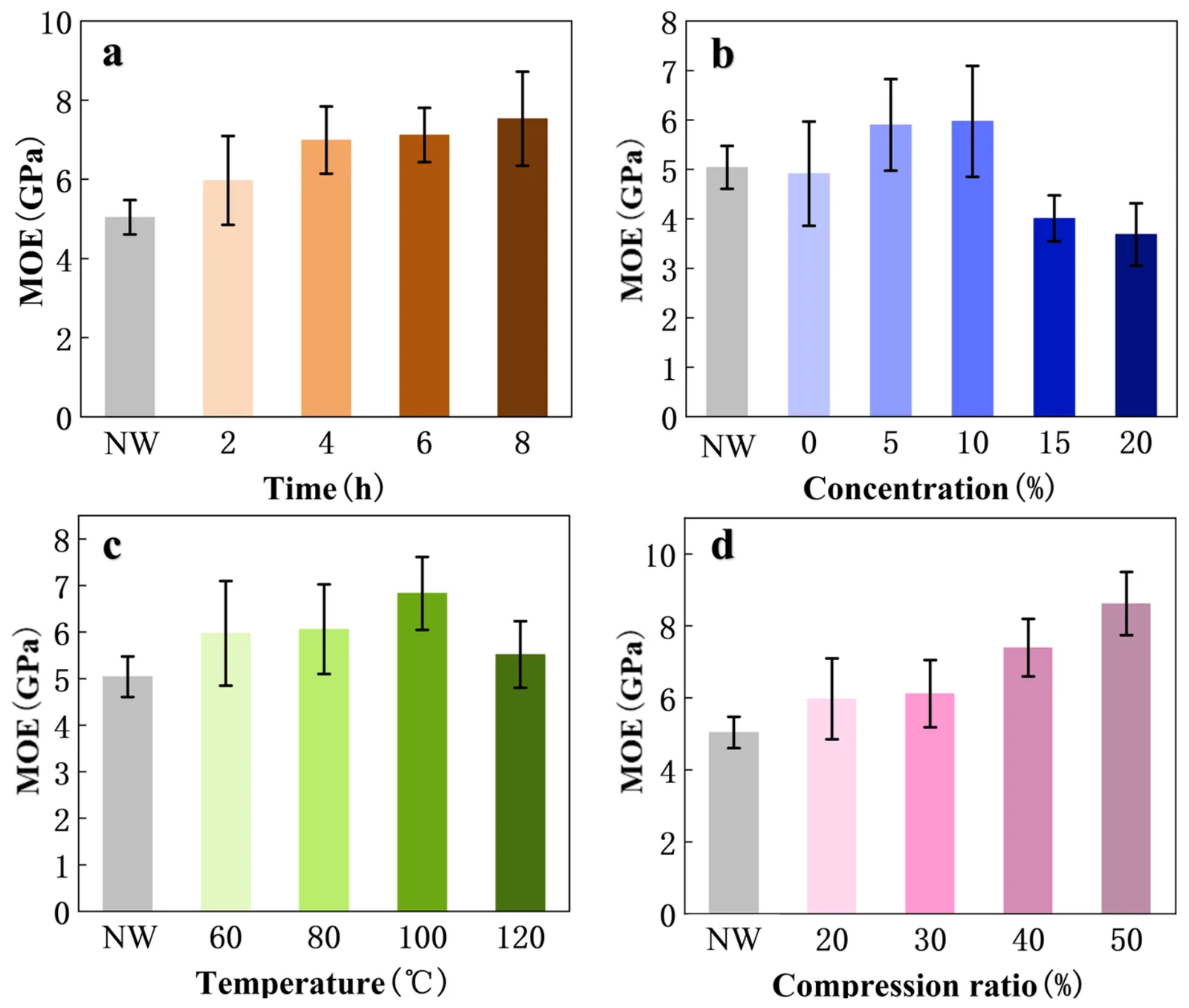
Modulus of elasticity of NW and DCF at different (**a**) treatment times, (**b**) DES concentrations, (**c**) treatment temperatures, and (**d**) compression ratios. (Note: Error bars represent standard deviations).

**Figure 4:**
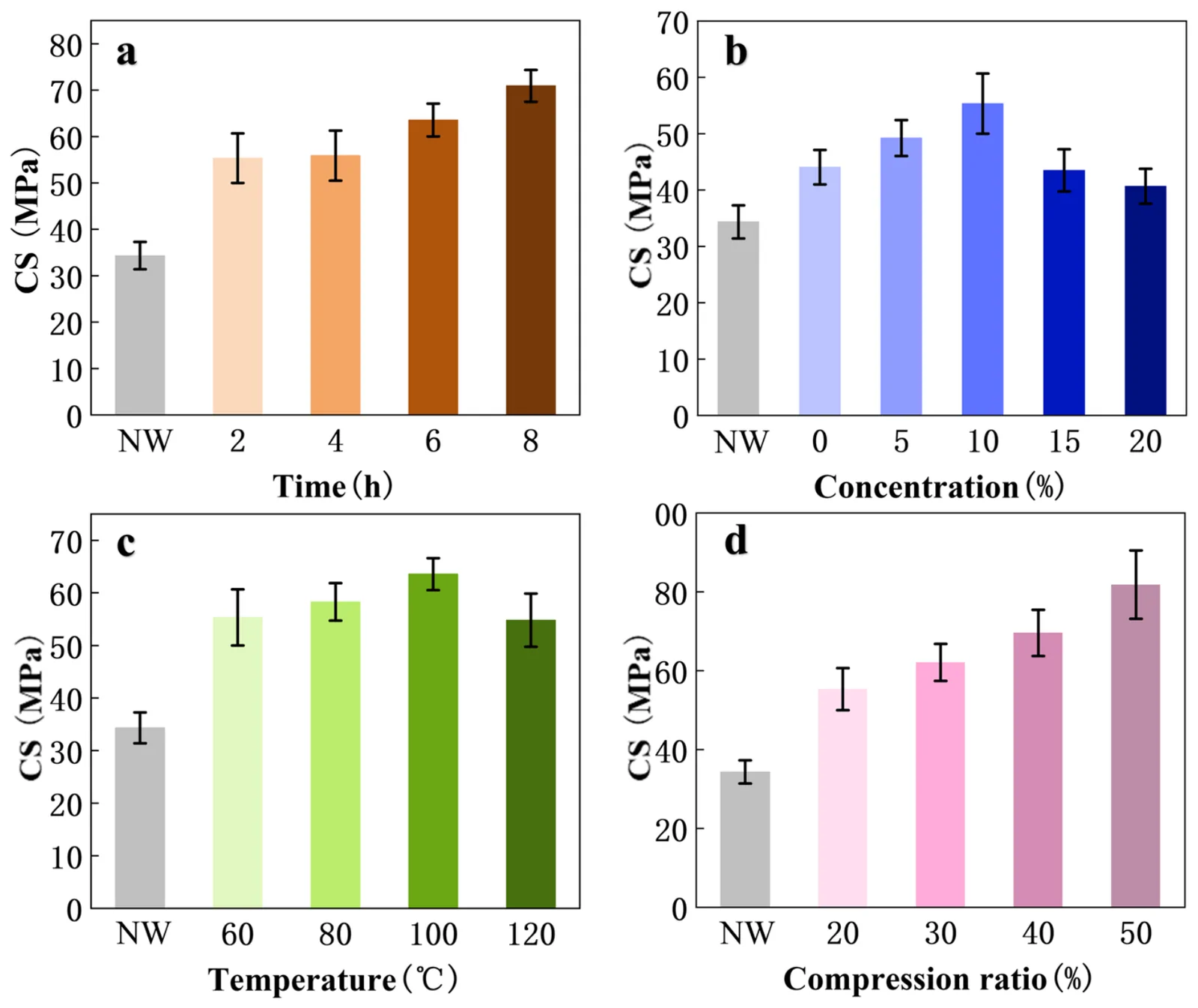
Compressive strength of NW and DCF at different (**a**) treatment times, (**b**) DES concentrations, (**c**) treatment temperatures, and (**d**) compression ratios. (Note: Error bars represent standard deviations).

**Figure 5:**
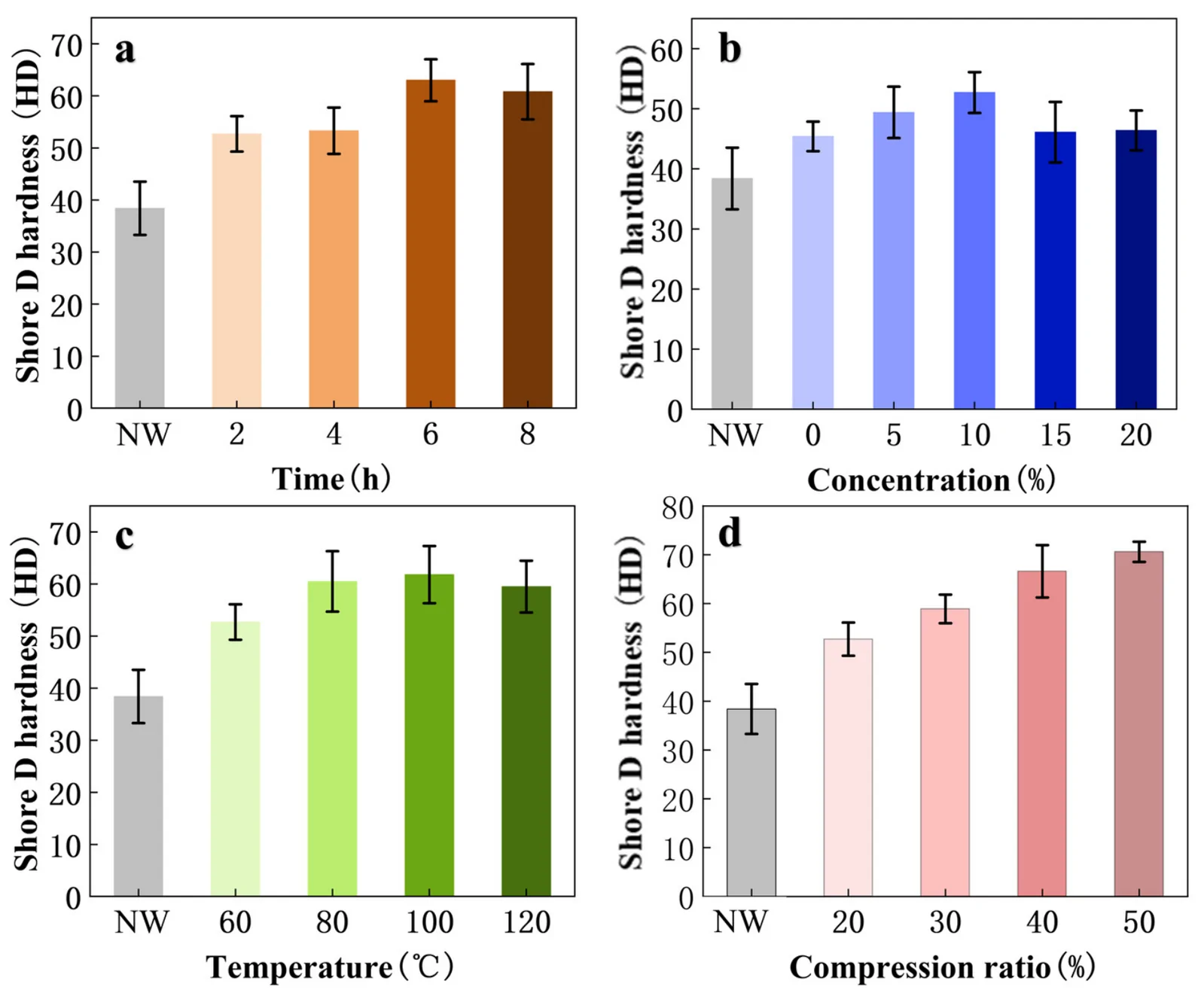
Surface Shore D hardness of NW and DCF at different (**a**) treatment times, (**b**) DES concentrations, (**c**) treatment temperatures, and (**d**) compression ratios. (Note: Error bars represent standard deviations).

Compared with NW, at a 50% compression ratio, the modulus of rupture of DCF increased from 56.1 MPa to 147.8 MPa, reflecting a 163.5% increase (Figure 2); the modulus of elasticity increased from 5040.5 MPa to 8622 MPa (Figure 3). The compressive strength parallel to the grain increased from 34.3 MPa to 81.8 MPa (Figure 4); and the surface Shore D hardness increased from 38.4 to 70.6 (Figure 5). As shown in the results, this comprehensive improvement in mechanical properties is primarily attributable to the substantial reduction in porosity following hot pressing, the increase in the number of cell walls per unit cross-sectional area, the densification of the cell walls in the surface and core layers, and the substantial enhancement of the inter-fiber contact area and mechanical interlocking [25].

Both DES concentration and pretreatment temperature exhibited a trend of initially increasing and then decreasing with respect to mechanical properties (Figure 2b,c, Figure 3b,c, Figure 4b,c and Figure 5b,c). Based on a pretreatment at 60 °C for 2 h, the flexural strength increased from 73.2 MPa in the distilled water control group to 82.3 MPa after treatment with 10 wt% DES but decreased to 67.6 MPa under 20 wt% DES conditions. Furthermore, when the pretreatment temperature increased from 60 °C to 100 °C, the plastic deformation capability of the cell walls was greatly enhanced, resulting in increases in the flexural strength, elastic modulus, and hardness of DCF. Conversely, when the temperature was further raised to 120 °C, reductions in all three mechanical properties were observed to various degrees. In other words, there is a distinct process window for DES-assisted hot-pressing: insufficient plasticization hinders uniform folding of the cell walls, while excessive pretreatment weakens the intercellular bonding of the cellulose framework.

As is widely recognized, the compression ratio is one of the most critical physical factors determining the extent of improvement in mechanical properties. As depicted in Figure 2d and Figure 4d, both flexural strength and compressive strength along the grain direction increased markedly as the compression ratio rose from 20% to 50%, indicating that the densification of the samples gradually shifted from surface compaction to compaction throughout the entire thickness. Interestingly, DES pretreatment reduced the resistance of the cell walls to compressive deformation, allowing the samples to maintain a relatively intact tracheid fold structure even at high compression ratios (thereby preventing brittle fracture), resulting in a simultaneous increase in strength, stiffness, and surface hardness [8].

### 3.3. Dimensional Stability and Compression Shape Fixation Regulated by DES

**Figure 6:**
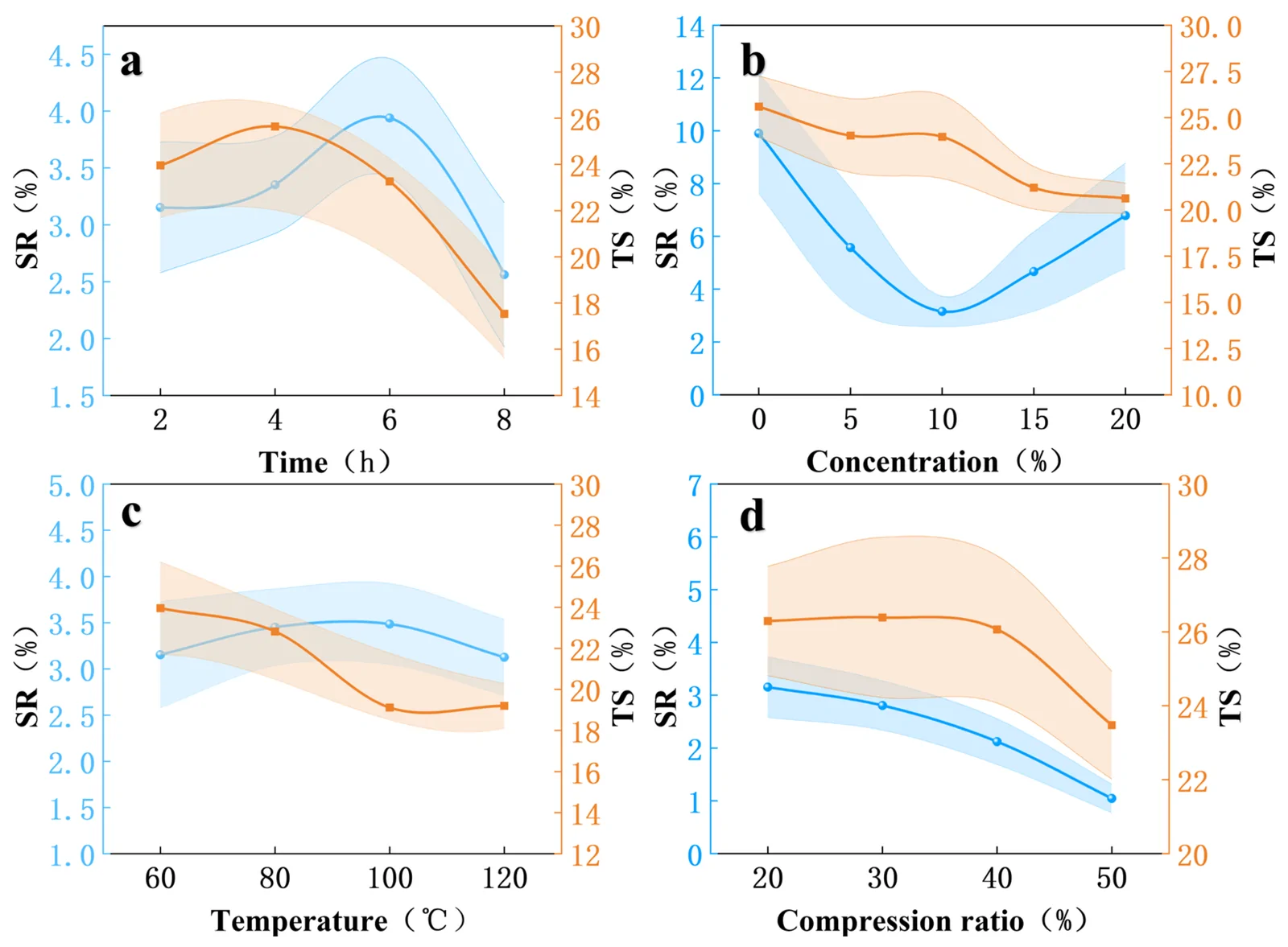
Moisture-induced rebound rate and thickness swelling of DCF at different (**a**) treatment times, (**b**) DES concentrations, (**c**) treatment temperatures, and (**d**) compression ratios.

Moisture-induced rebound is one of the primary failure mechanisms in compressed wood applications [7]. When moisture enters the cell wall, it breaks the hydrogen bonds between cellulose and hemicelluloses molecules, facilitating the release of elastic potential energy stored during compression, which in turn leads to a return to the original thickness [26]. Nevertheless, DES pretreatment can effectively improve shape retention by softening the cell walls, reducing resistance to compressive deformation, and promoting interface bonding after compression. Evidently, the DES concentration exhibited a distinct optimal range for moisture-induced rebound (Figure 6b): as the concentration increased from 0 to 10 wt%, the moisture-induced rebound rate decreased from 9.9% to 3.15%; further increases in concentration did not result in continued improvement in dimensional stability. These results demonstrate that moderate DES treatment facilitates the tight bonding of fiber bundles and hydrogen bond reconstruction, whereas excessive component removal may introduce structural defects and enlarge moisture transmission channels, which instead weakens stability.

Moreover, DES treatment time and temperature also greatly affect the fixation of the compressed morphology (Figure 6a,c). Specifically, longer treatment times facilitate the deep penetration of DES and the regulation of its components, while higher temperatures can effectively promote cell wall plasticization; however, extremely high temperatures will cause damage to the mechanical framework of the fibers. Comprehensively considering dimensional stability and macroscopic mechanical properties, the preparation process was determined to be: 10 wt% DES concentration, treatment at 100 °C for 8 h, and a 50% compression ratio (Figure 6d). Under these conditions, the moisture-induced rebound rate of DCF was as low as 0.77%, and the 24 h thickness swelling rate was only 6.31%, representing substantial reductions of 68.2% and 31.5%, respectively, compared to densified wood treated with pure water (Table 1). As such, DES-assisted hot-pressing densification can effectively lock in the compressed state recorded in the samples and effectively suppress thickness recovery in high-humidity environments [15].

### 3.4. Density Profile Distribution and Full-Thickness Densification

**Figure 7:**
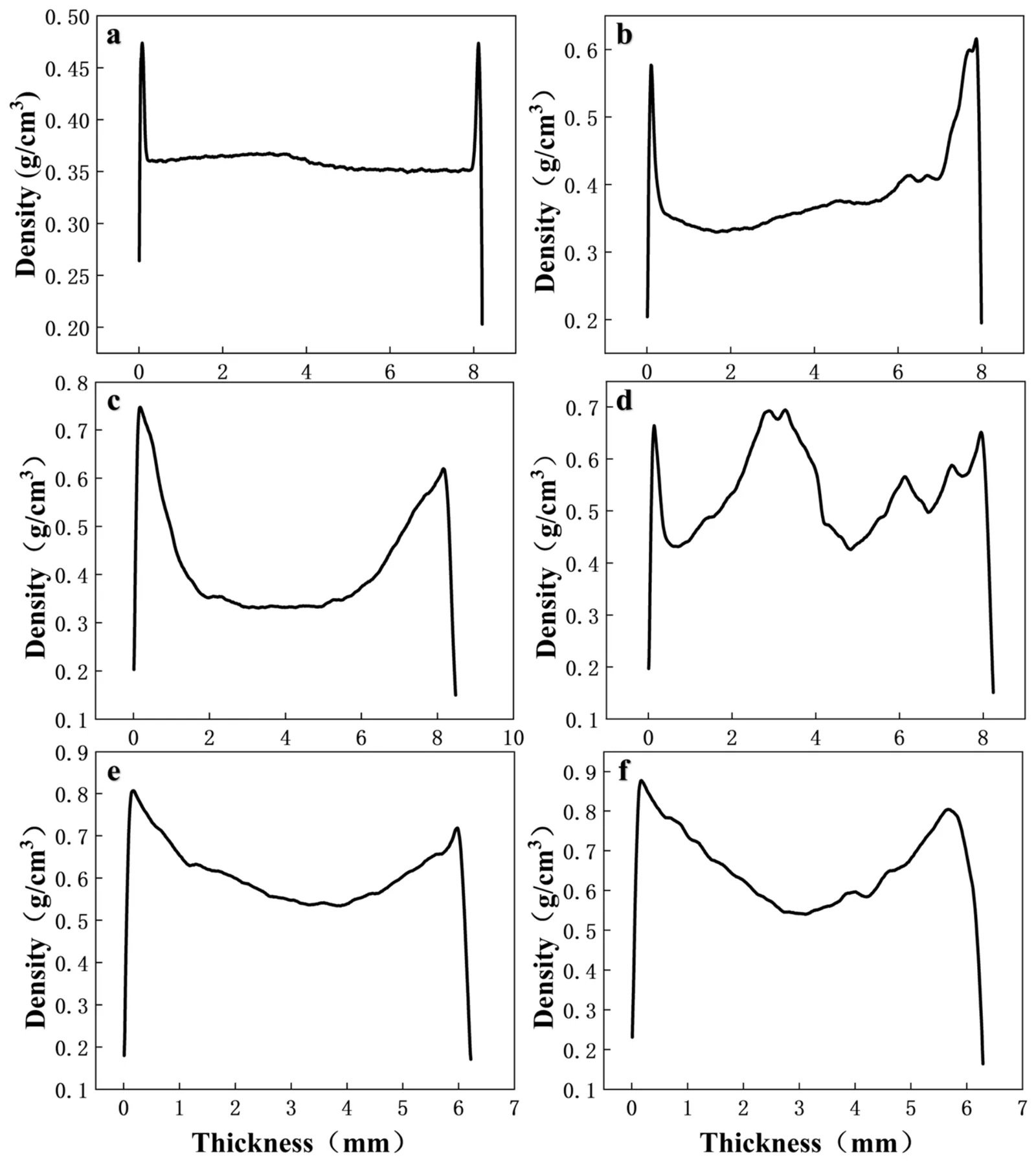
(**a**) Density profile of NW; (**b**) Density profile of pure water-treated densified Chinese fir; (**c**–**f**) Density profiles of DCF at compression ratios of 20, 30, 40, and 50%.

The density profile distribution allows for a visual assessment of whether compressive deformation is confined to the surface layer or extends throughout the entire thickness [3]. Evidently, the NW had a lower density that was distributed uniformly along the thickness (~0.35–0.37 g/cm^3^, Figure 7a). After DES pretreatment and hot-pressing, the average density of the samples showed a noticeable upward trend with increasing compression ratio (Figure 7c–f), reaching 0.42, 0.53, 0.60, and 0.65 g/cm^3^, respectively, representing increases of 16.62%, 47.93%, 67.50%, and 81.35% compared to the NW.

At low compression ratios, the density profile exhibited a U-shaped distribution (Figure 7c), indicating that the surface layer is highly densified, while the core layer retains a significant amount of porosity; in other words, weak zones are likely to form in the core layer under loading. After increasing the compression ratio to 30–40% (Figure 7d,e), the core layer density increased substantially, and the density profile tended to be uniform; when the compression ratio reached 50% (Figure 7f), the densified layer basically penetrated the entire thickness direction, and the core weakening problem was effectively alleviated. Accordingly, the improvement in mechanical properties originates not only from an increase in average density, but also from greater density uniformity in the thickness direction and enhanced continuity of load transfer [27].

### 3.5. Micro-Morphology and Flexible Cell Wall Folding

**Figure 8:**
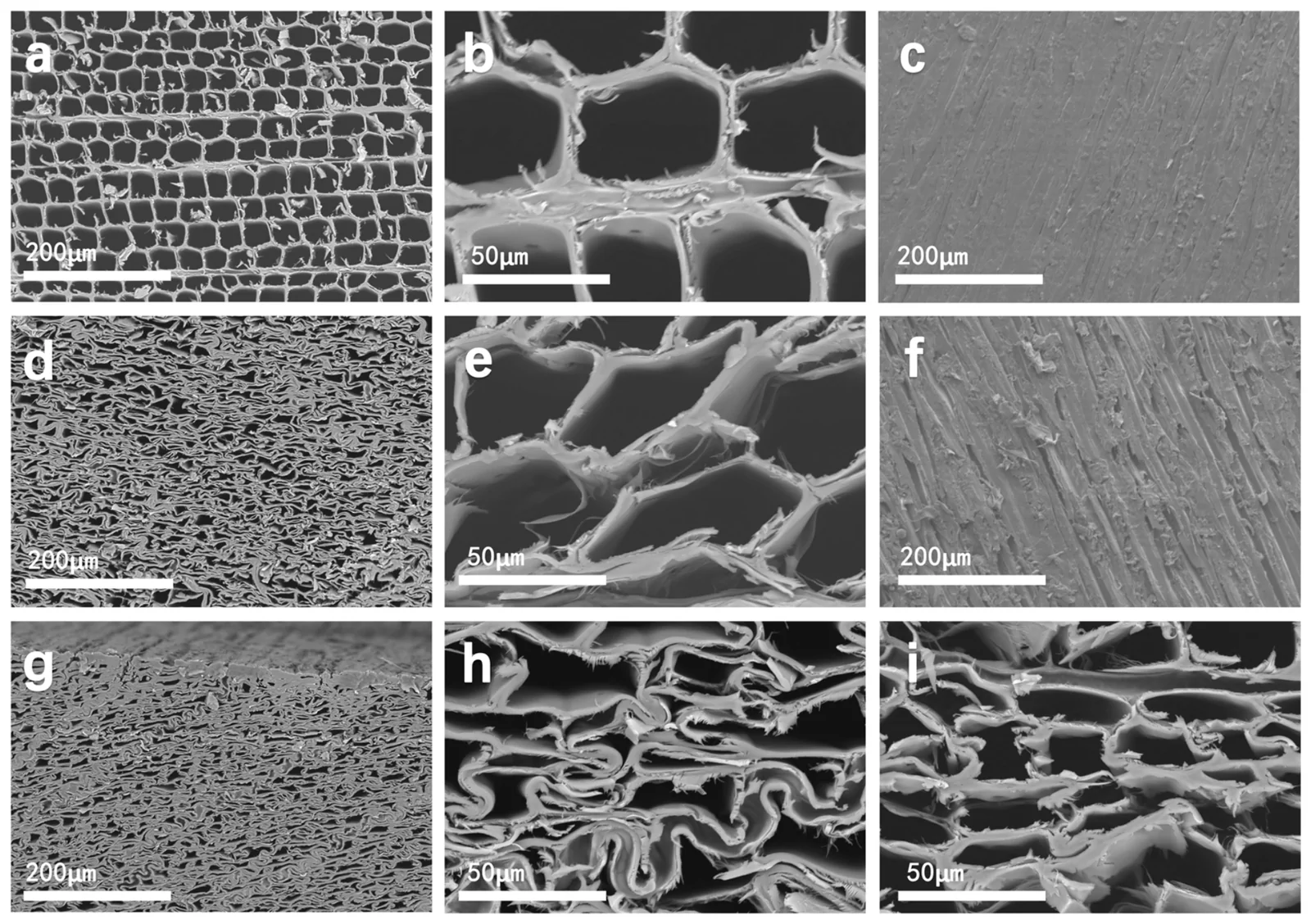
(**a**,**b**) SEM images of NW at different magnifications; (**c**) SEM image of the surface of NW; (**d**,**e**) SEM images of the sample at a 50% compression ratio at different magnifications; (**f**) SEM image of the surface of the DES-treated sample; (**g**) SEM image of the surface layer at a 50% compression ratio; (**h**) SEM image of the sample treated at 100 °C; (**i**) SEM image of the sample treated at 120 °C.

The transformation of Chinese fir from a loose, porous structure to a dense, load-bearing structure can be demonstrated using SEM. Apparently, NW exhibited a typical softwood structure, with intact tracheid cell lumina, thin cell walls, and interconnected pores (Figure 8a–c). After DES pretreatment, the surface roughness of the cell wall increased, and some fiber bundles were exposed (Figure 8f), indicating that the intercellular layer and the lignin–carbohydrate complex were weakened, resulting in reduced cell wall rigidity [13]. Consequently, this structural relaxation created conditions for flexible folding during the subsequent hot-pressing process.

At a 50% compression ratio, the cell cavities of the tracheids collapsed noticeably, and the cell walls folded and fit tightly together, but no large-scale delamination occurred in the overall structure (Figure 8d,e,g). These results indicated that DES pretreatment effectively reduced the cell wall deformation resistance, enabling the tracheids to achieve densification through flexible bending and surface contact rather than through brittle fracture [8]. Moreover, a comparison of different pretreatment temperatures revealed that at 100 °C, cell wall folding is continuous and exhibits fewer defects (Figure 8h), whereas at 120 °C, localized cracking, interlaminar separation, and irregular voids may occur (Figure 8i), indicating that excessive pretreatment can compromise cell wall integrity and weaken subsequent mechanical properties.

### 3.6. Changes in Chemical and Crystalline Structures Revealed by FTIR and XRD

**Figure 9:**
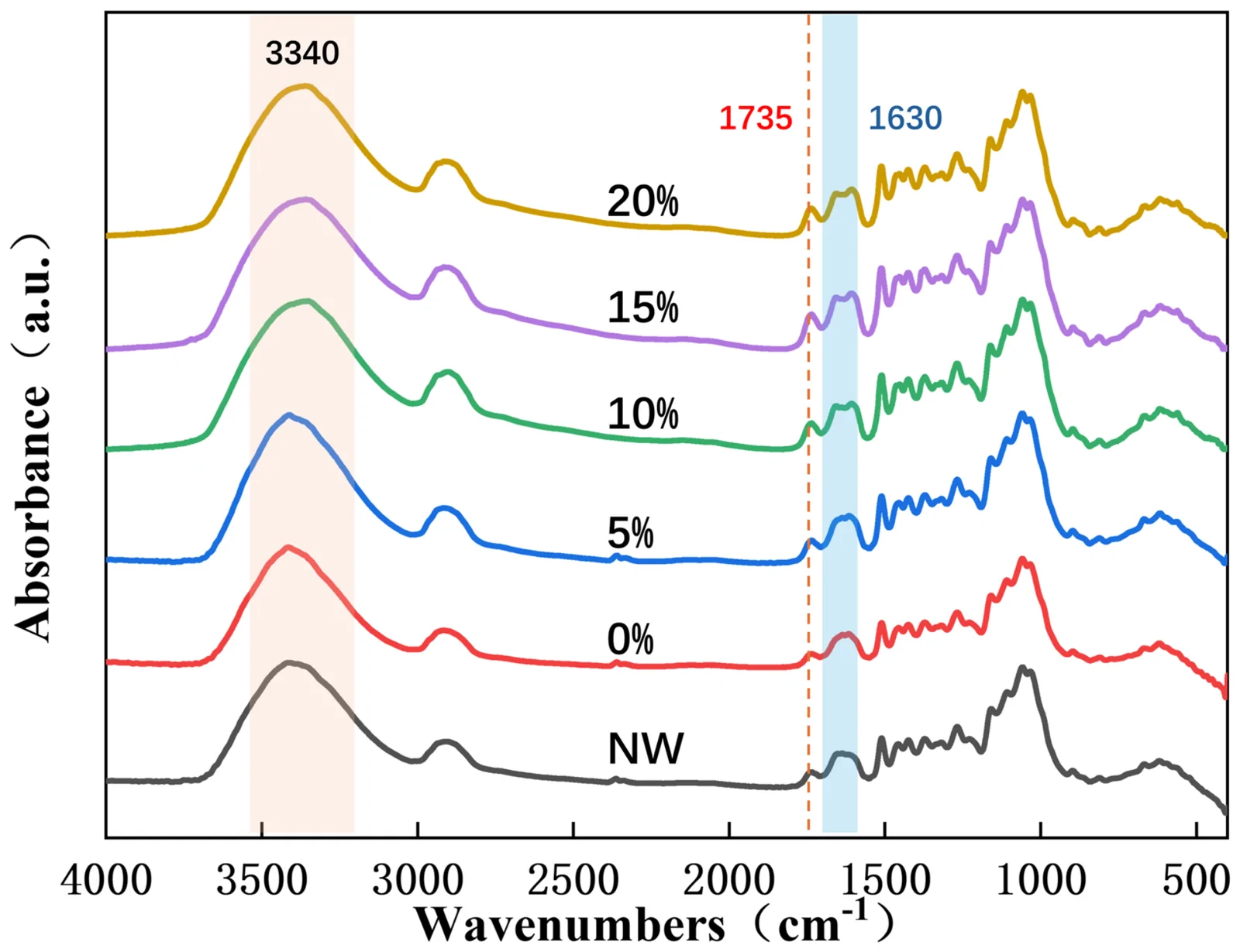
FTIR spectra of NW and DCF at different DES concentrations.

FTIR results (Figure 9) showed that DES pretreatment primarily acts on the amorphous regions rich in hemicellulose and lignin [28]. Additionally, the weakening of the C=O absorption peak at ~1730 cm^−1^ indicates partial removal of the acetyl or carbonyl groups from hemicelluloses; the minimal change in the vibrational peak of the lignin aromatic skeleton at ~1510 cm^−1^ suggests that the aromatic main chain of lignin was not substantially disrupted in this system. Both the C-O-C vibration near 1030 cm^−1^ and the broad O-H peak near 3340 cm^−1^ were relatively enhanced; this may be related to the increased relative content of celluloses, a potential reorganization of the hydrogen-bond network following densification, and the interaction between DES and the hydroxyl groups in the wood.

**Figure 10:**
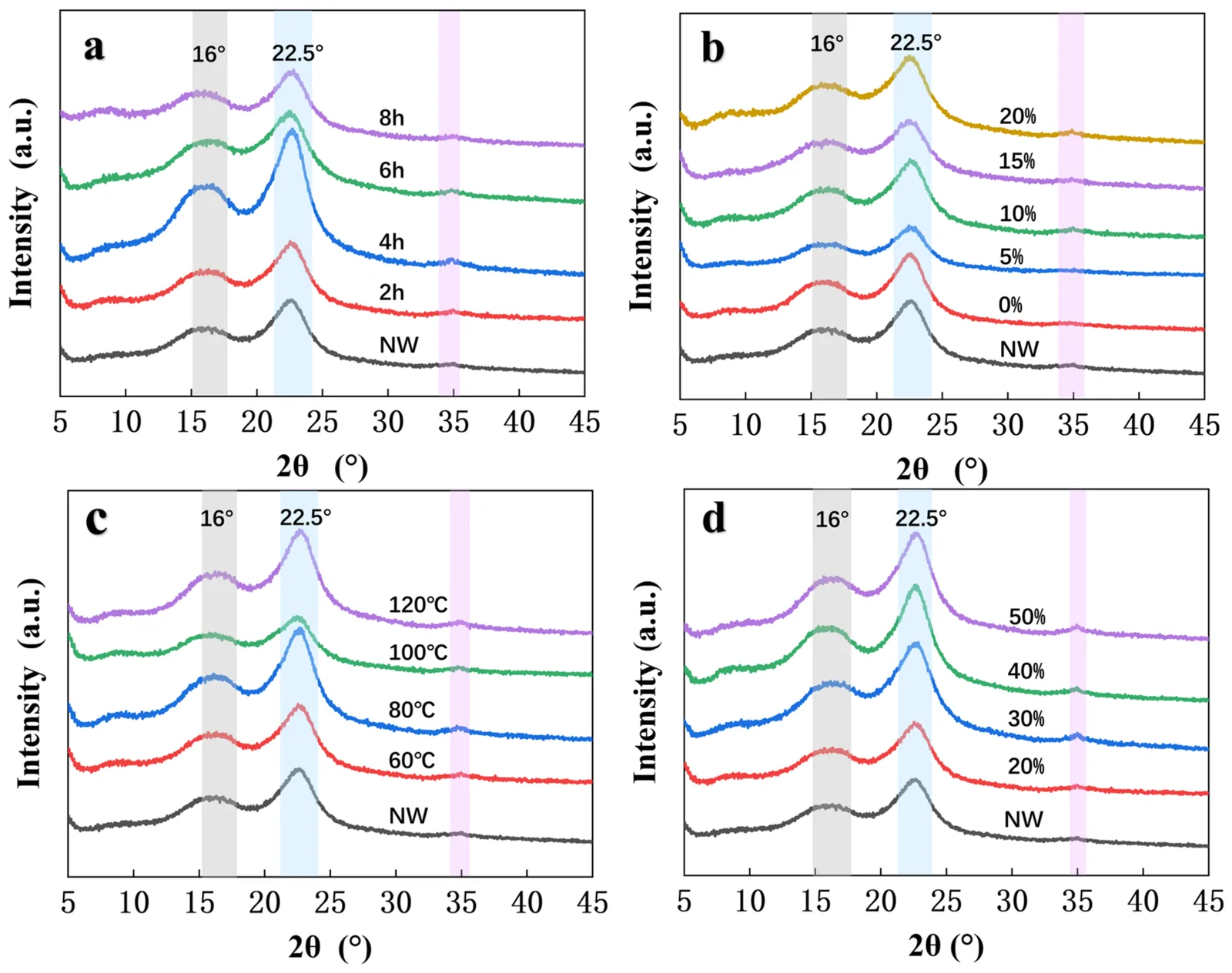
XRD patterns of NW and DCF at different (**a**) treatment times, (**b**) DES concentrations, (**c**) treatment temperatures, and (**d**) compression ratios. (The purple shadow indicates the characteristic diffraction peak near 2θ ≈ 34.5°).

The XRD patterns reveal that all samples retained characteristic cellulose I diffraction peaks near 2θ ≈ 16°, 22.5°, and 34.5°, indicating that DES pretreatment and hot-pressing largely maintained the crystalline form of cellulose [29]. Although a moderate reduction or adjustment of the crystalline/amorphous interface promotes cell wall plastic flow and pore closure, at high temperatures of 120 °C (Figure 10c), even though the relative crystallinity may increase due to the preferential degradation of the amorphous component [30], mechanical properties may still deteriorate as a result of an increase in cell wall defects and weakened interfacial bonding. As such, the essence of performance enhancement lies in the synergistic effect of chemical plasticization, preservation of the cellulose framework, macroscopic densification, and the reconstruction of hydrogen-bond/mechanical interlocking.

### 3.7. Rotary-Cut Surface Micro-Topography and Machining Adaptability

**Figure 11:**
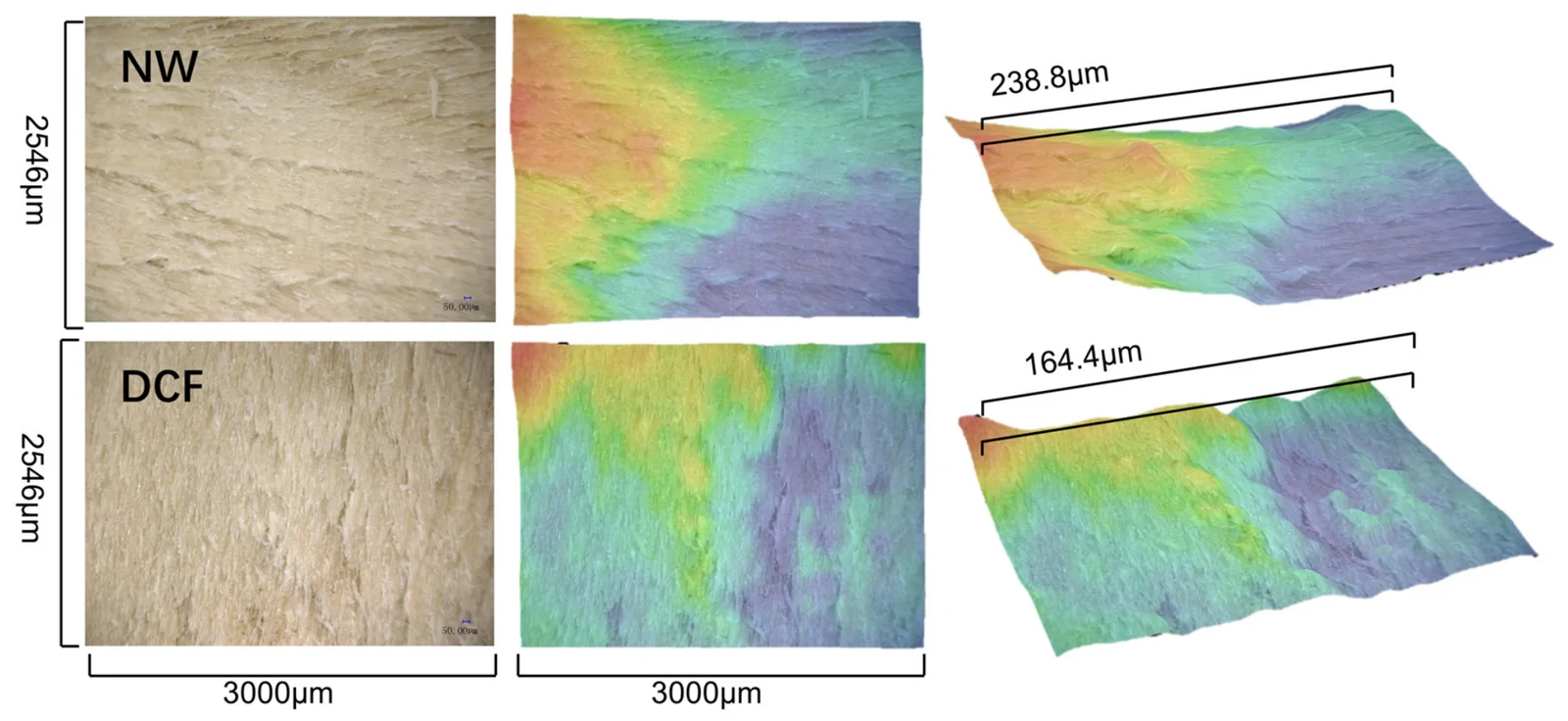
Three-dimensional deep-field microscopic morphologies of NW and DCF rotating cut surfaces. (The colors represent the surface height distribution).

For pencils, veneers, and precision-molded wood products, the surface quality of rotary-cut wood directly affects subsequent gluing, coating, and end-use performance. The rotary-cut interface of NW was rough, with noticeable peaks and valleys, and a maximum height difference of 238.8 μm (Figure 11). This is because NW has a loose structure, large tracheid lumina, and weak interfacial bonds, making it difficult for cutting stresses to be transmitted continuously during rotary cutting, which in turn can easily lead to fiber pull-out and irregular tearing of cell walls.

Remarkably, the maximum height difference on the DCF rotary-cut surface decreased to 164.4 μm, a reduction of ~31.2%; in other words, surface irregularities were markedly reduced. A possible explanation is as follows: DES plasticization and radial hot-pressing effectively compacted the hollow tracheids, resulting in cell walls that adhere closely together and form stronger microscopic mechanical interlocks, thereby facilitating the continuous transmission of cutting forces along the dense matrix and reducing fiber pull-out and tearing [12]. Accordingly, DES-assisted densification not only improves mechanical properties and dimensional stability but also enhances the ultimate processing suitability recorded for the samples.

### 3.8. Action Mechanism

The action mechanism of DES-assisted hot-pressing densification can be summarized into three stages. In the first stage, the DES of choline chloride and ethylene glycol penetrates the hierarchical pores of Chinese fir, likely weakening hemicelluloses, intercellular lignin, and lignin–carbohydrate complexes through hydrogen bond competition, polar swelling, and weak deconstruction, thereby imparting compressible plasticity to the cell walls under low mass-loss conditions [31]. In the second stage, the plasticized tracheid cell walls undergo flexible bending, collapse, and surface contact during the hot-pressing process at 140 °C, gradually forming a dense structure that extends from the surface layer to the core layer as the compression ratio increases. In the third stage, cooling under pressure and the close contact between cell walls are believed to promote the potential reorganization of hydrogen bonds and mechanical interlocking between cellulose-enriched surfaces, thereby locking in the compressed morphology and enhancing load-bearing capacity [14]. When the DES treatment is too intense, excessive removal of hemicelluloses/lignin and potential damage to cellulose can introduce microcracks and interfacial defects, leading to a reduction in strength and stability. Accordingly, the key to this process lies in controlling the degree of plasticization, ensuring it reduces compression resistance while maintaining the integrity of the cell wall skeleton. Despite the promising enhancements in the mechanical properties and dimensional stability of DCF, it should be objectively acknowledged that the current laboratory-scale process faces several sustainability and scalability challenges. Specifically, the process involves prolonged treatment times at elevated temperatures, repeated washing steps, and high-pressure hot-pressing, all of which consume considerable energy and water. Additionally, the recovery of DES from washing wastewater and its subsequent recycling have not yet been systematically resolved. Therefore, future research must focus on optimizing closed-loop solvent recycling systems, minimizing freshwater usage, and reducing overall energy consumption to facilitate the scalable, cost-effective, and genuinely sustainable industrial application of this densification strategy.

## 4. Conclusions

In summary, DCF was successfully prepared using sequential DES pretreatment followed by hot pressing. Given the mild nature of the DES treatment, the maximum mass-loss rate was 6.26%, which improved cell wall plasticity while preserving the overall wood framework. Moreover, the preferable preparation process was determined to be a DES concentration of 10 wt%, a treatment temperature of 100 °C, a treatment time of 8 h, and a compression ratio of 50%. It should be noted that these selected conditions are defined strictly within the investigated range, and the selection criterion was based on a comprehensive evaluation of achieving the highest mechanical properties and dimensional stability among the tested groups. The selected sample exhibited a modulus of rupture of 147.8 MPa, a modulus of elasticity in bending of 8622 MPa, a compressive strength parallel to the grain of 81.8 MPa, and a surface Shore D hardness of 70.6, representing increases of 163.5%, 71.1%, 138.5%, and 83.9%, respectively, compared to NW. Of particular note, the spectroscopic and structural analyses suggest that DES pretreatment partially modulates the amorphous fraction, potentially facilitates the reorganization of the hydrogen-bond network, and largely retains the cellulose I crystalline form. The improvement in the overall performance of the samples primarily results from the synergistic effects of low-loss plasticization, structural densification, preservation of the cellulose framework, and interfacial reorganization. This work aims to provide a feasible route for transforming fast-growing Chinese fir into high-value-added engineering lumber and precision-machined wood products.

## Figures and Tables

**Table 1:** Comparison of the mechanical properties of samples.

Properties	NW	DCF	Improvement/Results
Modulus of rupture (MPa)	56.1 ± 5.5	147.8 ± 18.2	+163.5%
Modulus of elasticity (MPa)	5040.5 ± 434.9	8622 ± 878.5	+71.1%
Compressive strength parallel to grain (MPa)	34.3 ± 2.9	81.8 ± 8.7	+138.5%
Surface Shore D hardness	38.4 ± 5.1	70.6 ± 2.1	+83.9%
Moisture-induced rebound rate, *SR* (%)	/	0.77 ± 0.06	Low rebound rate
24 h thickness swelling rate, *TS* (%)	/	6.31 ± 0.45	Improved dimensional stability
Maximum height difference of rotary-cut surface (μm)	238.8	164.4	−31.2%

## Data Availability

The original contributions presented in this study are included in the article. Further inquiries can be directed to the corresponding authors.
